# The Effect of FGF23 on Cardiac Hypertrophy Is Not Mediated by Systemic Renin-Angiotensin- Aldosterone System in Hemodialysis

**DOI:** 10.3389/fmed.2022.878730

**Published:** 2022-04-26

**Authors:** Katharina Dörr, Michael Kammer, Roman Reindl-Schwaighofer, Matthias Lorenz, Rodrig Marculescu, Marko Poglitsch, Dietrich Beitzke, Rainer Oberbauer

**Affiliations:** ^1^Department of Nephrology, Medical University of Vienna, Vienna, Austria; ^2^Center for Medical Statistics, Informatics, and Intelligent Systems, Section for Clinical Biometrics, Medical University of Vienna, Vienna, Austria; ^3^Vienna Dialysis Center, Vienna, Austria; ^4^Department of Laboratory Medicine, Medical University of Vienna, Vienna, Austria; ^5^Attoquant Diagnostics, Vienna, Austria; ^6^Department of Biomedical Imaging and Image-Guided Therapy, Division of Cardiovascular and Interventional Radiology, Medical University of Vienna, Vienna, Austria

**Keywords:** renin-angiotensin-aldosterone-system, FGF23, hemodialysis, left ventricular hypertrophy, chronic kidney disease

## Abstract

Fibroblast growth factor 23 (FGF23) is elevated in patients with chronic kidney disease and contributes to left ventricular hypertrophy (LVH). The aim of the analysis was to determine whether this effect is mediated by the renin-angiotensin-aldosterone system (RAAS) in hemodialysis. Serum samples from 62 randomized hemodialysis patients with LVH were analyzed for plasma renin activity (PRA-S), angiotensin II (AngII), and metabolites, angiotensin-converting enzyme-2 (ACE2) and aldosterone using a high throughput mass spectrometry assay. Compared to healthy individuals, levels of the RAAS parameters PRA-S, AngII and aldosterone were generally lower [median (IQR) PRA-S 130 (46–269) vs. 196 (98, 238) pmol/L; AngII 70 (28–157) vs. 137 (76, 201) pmol/L; Aldosterone 130 (54, 278) vs. 196 (98, 238) pmol/L]. We did not find an indication that the effect of FGF23 on LVH was mediated by RAAS parameters, with all estimated indirect effects virtually zero. Furthermore, FGF23 was not associated with RAAS parameter levels throughout the study. While there was a clear association between FGF23 levels and left ventricular mass index (LVMI) at the end of the study and in the FGF23 fold change and LVMI change analysis, no association between RAAS and LVMI was observed. Serum concentrations of PRA-S, AngII, and aldosterone were below the ranges measured in healthy controls suggesting that RAAS is not systemically activated in hemodialysis patients. The effect of FGF23 on LVMI was not mediated by systemic RAAS activity. These findings challenge the current paradigm of LVH progression and treatment with RAAS blockers in dialysis.

**Clinical Trial Registration:**

[https://clinicaltrials.gov/ct2/show/NCT03182699], identifier [NCT03182699].

## Introduction

The renin-angiotensin-aldosterone system (RAAS) is a neurohumoral signaling pathway playing a central role in the maintenance of the blood volume, vascular tone and water homeostasis in the kidney (1, 2). Studies in patients without chronic kidney disease (CKD) showed that chronic activation of the systemic RAAS causes left ventricular hypertrophy (LVH) via volume expansion and direct effects of angiotensin II (AngII) on the myocardium (3). RAAS blocking medication is known to improve LVH and reduce cardiovascular risk in the general population (4).

In CKD the concentration of Fibroblast growth factor 23 (FGF23) rises with decreasing glomerular filtration rate, and high FGF23 was shown to stimulate cardiomyocyte enlargement (5, 6). So far it is known that the promotion of LVH by FGF23 could be induced via a direct activation of Fibroblast growth factor receptor 4 on cardiac myocytes as well as through FGF23 induced volume expansion and hypertension (6). Another pathway of FGF23 induced cardiac hypertrophy, which is discussed by the scientific community, is via an induction of sodium and calcium retention, leading to volume expansion and hypertension (7, 8). Animal models showed that FGF23 may exert a direct stimulatory effect on the RAAS through the suppression of angiotensin-converting enzyme-2 (ACE2) (9). The latter converts AngII to Ang(1-7), therefore counteracting its effects and influencing the blood pressure regulation and heart and endothelial function (10–13). ACE2 overexpression ameliorates LV-remodeling and LV-dysfunction (14). The suppression of ACE2 by FGF23 suggests an alternative additional mechanism possibly explaining the accelerating effect of FGF23 on LVH. Moreover, FGF23 induced calcitriol suppression indirectly upregulates renin production in the kidney, activating the RAAS-cascade at its beginning (15).

We previously demonstrated an association of FGF23 and aldosterone serum levels in CKD in humans and animals, providing additional evidence for a crosstalk between FGF23 and RAAS parameters (16). Furthermore, we recently showed in a randomized controlled trial, that calcimimetic treatment with etelcalcetide (ETL) suppressing FGF23 could inhibit the progression of LVH in hemodialysis (17). In the present study we sought to evaluate whether this FGF23 suppression by ETL and its effect on LVH in dialysis patients was mediated by members of the RAAS cascade (18, 19).

## Materials and Methods

### Study Objective, Design, and Participants

**FIGURE 1:**
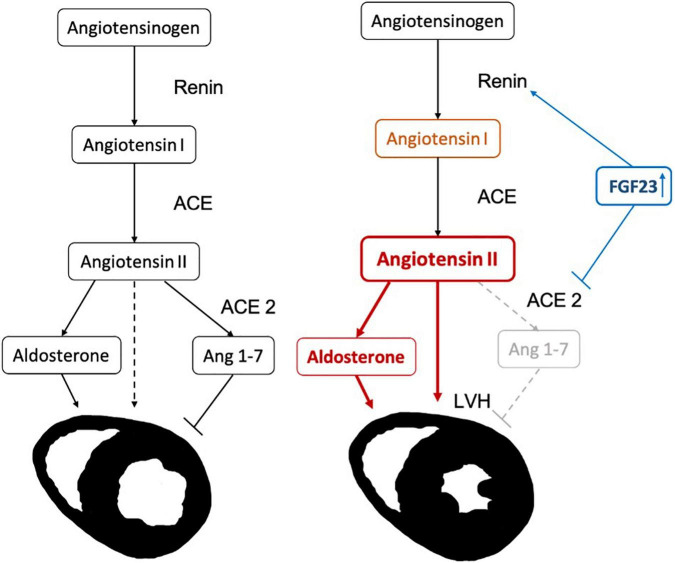
Hypothesis for the progression of left ventricular hypertrophy (LVH) due to FGF23 induced activation of the renin-angiotensin-aldosterone system (RAAS) cascade: FGF23 increases renin (indirectly by reducing the levels of active vitamin D). In addition, the levels of ACE2 are inhibited, increasing AngII and decreasing Ang(1-7), causing an increase in left ventricular mass.

The primary objective of the study was to elucidate, whether the effect of FGF23 on LVH in patients on maintenance hemodialysis treatment was mediated by RAAS parameters. The key hypothesis is visualized in Figure 1 to facilitate understanding of the study motivation.

The ETECAR-HD study (Effect of etelcalcetide on cardiac hypertrophy in hemodialysis patients) was a prospective, randomized, controlled, single-blinded trial of maintenance hemodialysis patients with secondary hyperparathyroidism and LVH (20). Sixty-two patients were randomized in a 1:1 ratio to intravenous ETL or intravenous alfacalcidol for of one year. Left ventricular mass index (LVMI) was measured at baseline and after 1 year with cardiac magnetic resonance imaging (CMR). Data from 16 healthy controls served as reference. The trial was approved by the ethics committee of the Medical University of Vienna (MUV; [EK # 1127/2017]), the national regulatory authorities (AGES # 10087746) and conducted in accordance with the principles of the Declaration of Helsinki.

### Laboratory Analysis

Equilibrium concentrations of Ang I, Ang II, and Ang 1-7 as well as of the steroid aldosterone were measured in human serum by LC-MS/MS (Attoquant Diagnostics, Vienna, Austria) using previously validated and described methods (21, 22). Samples were collected on the first as well as the last day of the year-long treatment, or in case of drop-out, on the last study visit before drop-out, prior to heparin application and dialysis treatment, in a supine position. Probes were centrifuged at room temperature and frozen and stored at −80°C until RAAS equilibrium analysis. Briefly, following equilibration samples were stabilized and spiked with C13/N15 labeled internal standards for individual angiotensin metabolites and a deuterated internal standard for aldosterone. Extracted samples were concentrated by evaporation, reconstituted and analyzed using mass spectrometry analysis using a reversed- analytical column (Acquity UPLC C18, Waters) operating in line with a XEVO TQ-S triple quadrupole mass spectrometer (Waters Xevo TQ/S, Milford, MA, United States). The activity of the RAAS was estimated by using PRA-S, a clinically validated angiotensin based marker for plasma renin activity that is obtained by summarizing equilibrium levels of Ang I and Ang II (23, 24). Briefly, PRA-S has been shown to strongly correlate with classical methods to determine plasma renin activity (PRA) and plasma renin concentration (PRC). ACE2 activity was determined by an LC-MS/MS based AngII-to-Ang1-7 conversion assay using internal standardization with stable isotopes. ACE2 concentration was obtained through calibration with recombinant human ACE2. All RAAS component assays were successfully validated according to the EMA guideline for industry. The between-run CV at 15 pg/ml for Ang I, Ang II, Ang 1-7, Aldosterone and PRA-S were 6.7, 6.0, 7.2, 6.6, and 8.2%, respectively. LLOQs for Ang I, Ang II, Ang 1-7, Aldosterone and PRA-S were 3, 2, 3, 20, and 5 pmol/L, respectively. Active ACE2 concentration showed an LLOQ of 0.5 ng/ml with a between-run CV of 4%. Intact FGF23 levels were measured using a chemiluminescent immunoassay (DiaSorin^®^, measurement batched, duplicate, CV ≤ 3,8%).

### Study Definitions and Outcomes

Our primary outcomes was to assess if the effect of FGF23 on LVH was mediated by the RAAS parameters ACE2, AngII, and aldosterone.

### Statistical Analysis

All continuous variables were reported by median and interquartile range (i.e., 3rd to 4th quartile), categorical variables were reported by absolute and relative frequencies. We estimated individual FGF23 fold changes per year by a linear mixed effect model to incorporate all longitudinal FGF23 measurements. The model used log2-transformed FGF23 levels as outcome, and measurement time as covariate, as well as a random intercept and random effect of time per individual. Estimates of FGF23 fold change per year per individual were obtained by summing the fixed effect estimate of time and the random effect of time per individual. Model fit was assessed using residual analysis and the conditional R^2^. To investigate our study hypotheses, we used linear regression models. We assessed our primary hypotheses using the product method for mediation (25). To this end, we fitted two models: one for the change in LVMI including the change in single RAAS parameters as mediator and FGF23 fold change as main exposure, and one for the corresponding RAAS parameter including FGF23 fold change as exposure. The direct effect of FG23 on LVMI change is given as the effect estimate of FGF23 in the first model, while the indirect effect is given as the product of the effect estimates from both models. These models also used the randomization factors (study center, residual kidney function), as well as corresponding baseline RAAS and FGF23 levels as covariates, similar to our main analysis (17).

To study the results in more detail, we investigated the associations at baseline and end of study between levels of FGF23 and RAAS, as well as levels of FGF23, RAAS, and LVH, using linear regression models. First, to assess the association of levels at baseline, we fitted models for the baseline RAAS levels of each of the RAAS parameters of interest (ACE2, AngII, aldosterone) using FGF23 levels at baseline as main exposures. Second, we modeled the RAAS levels at the end of study using FGF23 fold change as main exposure. These models also used the corresponding baseline RAAS and FGF23 levels as covariates. To assess the contribution of FGF23 fold change per year to the overall explained variability of a model we computed the drop-in-R^2^ when FGF23 was removed from the model. Third, we fitted models for levels of LVMI at baseline and end of study using levels of a single RAAS parameters, or FGF23, as main exposures. For the end of study outcome, the baseline LVMI was used as additional covariate. Fourth, we fitted models for LVMI change using change in a single RAAS parameter, or FGF23 fold change, as main exposures. In all models using LVMI change as outcome, we used the same adjustment set as in the mediation analysis, namely the randomization factors and baseline levels of LVMI, the RAAS parameter and FGF23. To assess the impact of RAAS inhibiting medication, we conducted sensitivity analyses for all models by adding an interaction with a covariate representing the intake of RAAS medication (ACE inhibitors, AT2 blockers, Aldosterone antagonists) at baseline or during the study.

We checked model fit using residual diagnostics. All levels (FGF23 and RAAS outcomes) were log2-transformed, such that regression coefficients are to be interpreted as follows: if the exposure doubles in value, then the outcome is multiplied by the factor 2^coefficient.

PRA-S was not analyzed separately because of its derivation from AngI and II. All RAAS levels below the lower limit of quantification (LLOQ) were set to half the LLOQ. To assess the impact of this imputation procedure, we re-fitted all models on data excluding values below the LLOQ. As the amount of data missing due to other causes was low, we conducted complete case analyses for all outcomes. To account for the estimation of the FGF23 fold change via mixed models, we derived confidence intervals (CI) for estimates involving FGF23 fold changes via the percentile method on 1,000 bootstrap resamples. In each resample, the FGF23 fold change was re-estimated and all models re-fitted. No correction for multiple testing was applied to the reported CIs due to the exploratory nature of the study. The R statistical software version 4.0.2 was used for the analysis.

### Sample Size Considerations

The trial was initially powered for the analysis of the effect of the study drug on change in LVMI (17). For the present analysis, we computed that a study with 62 participants has 80% power to detect an effect size of 0.362 for a normally distributed exposure (i.e., the estimated FGF23 fold change) in a linear regression using a t-test with a two-sided significance level of 0.05 (nQuery software version 8). Assuming a standard deviation of 2 for the exposure, this translates as follows to the different primary outcomes: for ACE2 (log2-levels standard deviation around 0.7) a regression coefficient of around 0.13 and for AngII and aldosterone (log2-levels standard deviation around 2) regression coefficients of around 0.36 are detectable with 80% power, respectively.

## Results

### Participants and Laboratory/Renin-Angiotensin-Aldosterone System Parameters

**FIGURE 2:**
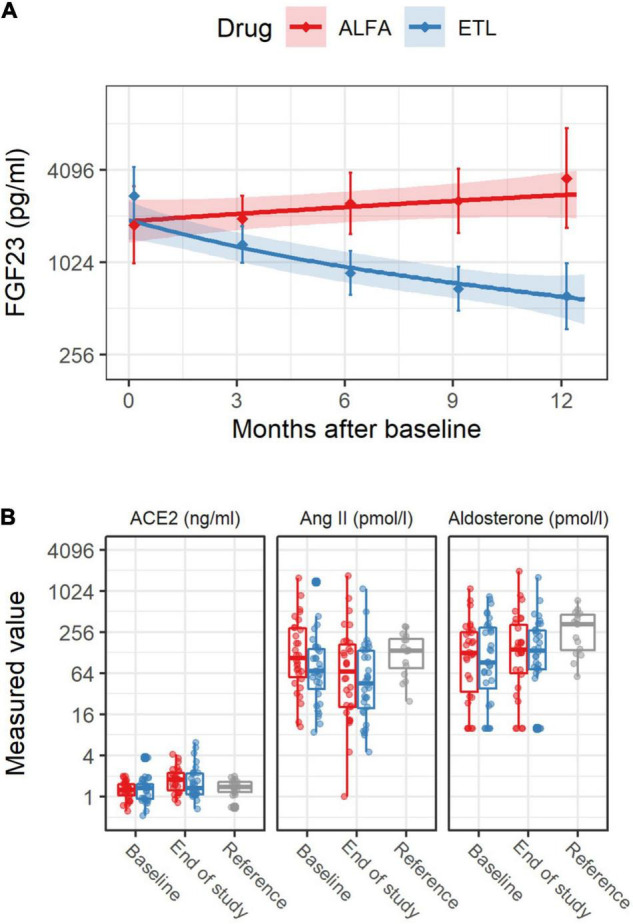
FGF23 levels [upper panel **(A)**] and renin-angiotensin-aldosterone system (RAAS) levels [lower panel **(B)**] over the study period, stratified by study drug. In panel **(A)**, the solid line was derived from a linear regression model for the log2 transformed FGF23 levels including measurement time as covariate. The shaded area depicts pointwise 95% confidence intervals for the predicted means at the given points in time. The diamonds and vertical bars at time 0 depict median and interquartile range for baseline FGF23 levels. The corresponding diamonds and vertical bars at later times summarize values from baseline to 3 months, from 3 to 6 months, and so on. In the panel **(B)** for RAAS levels depicted by boxplots, “Reference” refers to the RAAS values from a group of healthy male individuals, which were not part of this study, but are shown for comparison. Individual measurements are drawn as points.

A total of 62 patients were enrolled in the trial and randomized to receive ETL (*n* = 32) or alfacalcidol (*n* = 30) treatment. Fifty two patients successfully completed the whole trial as planned. However, in case of drop-out a RAAS analysis was performed from the probes collected on the last study visit before drop-out. Patient characteristics at baseline are reported in Table 1. We reported the inclusion and exclusion criteria in the data supplement of our previously published paper (17). Treatment groups showed no difference between systolic and diastolic blood pressure or the number of blood pressure medications. The use of RAAS-blocking medication, which did not change markedly during the study, is reported in Table 1. In total, 14.1 and 16.1 patient years with RAAS-blocking medication were accumulated over the whole study period for the ALFA and ETL groups, respectively. 84% of patients were under beta-blocker therapy, with no differences between study-treatment allocation. Table 2 gives numerical summaries for the levels of LVMI, FGF23, and the RAAS parameters at baseline and the end of study, including reference ranges of healthy volunteers for comparison. In median, nine longitudinal measurements for FGF23 were available per individual. Changes of levels of FGF23 were strongly influenced by the administered study drug, as shown in Figure 2A. The estimated FGF23 fold changes per year provided a precise summary of the individual FGF23 trajectories throughout the study, as depicted in Supplementary Figure 1, with a conditional R^2^ for the mixed model of 0.87 (95% CI from bootstrap 0.85–0.89). Levels of the RAAS parameters, in particular AngII and aldosterone, were generally lower in our study cohort than in the healthy control group, but quite comparable between both study drugs, as depicted in Figure 2B. Levels of AngII and aldosterone showed high inter-individual heterogeneity, and around 16% of the individuals had levels below the LLOQ for aldosterone. Measurements for Ang(1-7) were below the LLOQ for most individuals (72%). Such a small number of numerical measurements available would lead to an increased variability of all results concerning this RAAS parameter. Therefore, Ang(1-7) was removed from further analysis.

**TABLE 1:** Patient characteristics at baseline.

	Overall	Alfacalcidol	Etelcalcetide
Number of individuals	62	30	
Age (years), median [IQR]	62 [54, 68]	62 [54, 66]	66 [53, 71]
Sex (female)	16 (26%)	6 (20%)	10 (31%)
BMI, median [IQR]	27 [24, 30]	27 [24, 29]	28 [24, 33]
Dialysis duration before first application of study drug (months), median [IQR]	12 [5, 20]	12 [5, 23]	11 [4, 19]
Overhydration measured by BCM before hemodialysis (% of body weight), median [IQR]	2.2 [1.0, 3.9]	2.3 [1.0, 4.2]	2.1 [1.0, 3.8]
Mean arterial pressure, median [IQR]	96 [87, 101]	92 [85, 101]	98 [92, 102]
**Randomization factors**			
Dialysis center (MUV)	20 (32%)	10 (33%)	10 (31%)
Residual kidney function (≥500 ml/day)*	50 (81%)	24 (80%)	26 (81%)
**RAAS-blocking medication**			
ACE inhibitor therapy	6 (10%)	2 (7%)	4 (12%)
AT2 blocker therapy	21 (34%)	9 (30%)	12 (38%)
Aldosterone antagonist therapy	4 (7%)	3 (10%)	1 (3%)
**Comorbidities**			
Hypertension	61 (98%)	29 (97%)	32 (100%)
Hyperlipidemia	29 (47%)	14 (47%)	15 (47%)
Diabetes mellitus	26 (42%)	12 (40%)	14 (44%)
Peripheral vascular disease	13 (21%)	6 (20%)	7 (22%)
**Cause of end-stage kidney disease**			
Diabetic or vascular nephropathy	34 (55%)	17 (57%)	17 (54%)
ADPKD	10 (16%)	6 (20%)	4 (13%)
Glomerulonephritis	6 (10%)	2 (7%)	4 (13%)
Other	12 (19%)	5 (16%)	7 (21%)

*Continuous variables are described median and 1st–3rd quartile (interquartile range, IQR), categorical ones by absolute and relative frequencies. Age and residual kidney function were determined at the initial screening visit before randomization.*

*Urine output for assessment of residual kidney function is based on patient self-report and is routinely documented by nursing staff every 3 months for the calculation of the Kt/V. Patients were additionally questioned about their urine output at screening.

**TABLE 2:** Measurement data at baseline and end of study (after 1 year of follow-up or last measurement available, in case a patient dropped out).

	Baseline	End of study	Reference range
Left ventricular mass index (g/m^2^), median [IQR]	69 [62, 84]	72 [62, 87]	58 (11)^†^
FGF23 (pg/ml), median [IQR]	2,306 [858, 5,087]	1,064 [277, 3,750]	23–95
**RAAS parameter**			
PRA-S (pmol/L), median [IQR]	160 [75, 361]	115 [27, 240]	196 [98, 238]
ACE2 (ng/ml), median [IQR]	1.3 [0.9, 1.5]	1.5 [1.1, 2.2]	1.4 [1.2, 1.7]
Ang(1-7) (pmol/L)*	<3 [<3, 4]	<3 [<3, 4.4]	<3
Angiotensin II (pmol/L)	84 [45, 175]	53 [20, 141]	137 [76, 201]
Aldosterone (pmol/L)	122 [34, 271]	140 [71, 286]	196 [98, 238]

*Variables are described by median and 1st–3rd quartile (interquartile range, IQR).*

*Values below limit of quantification were set to half the lower limit of detection, for details see Supplementary Table 3. Missing values at baseline/end of study: FGF23 0/3, ACE2 2/5.*

*The comparison ranges were derived from the literature for LVMI by applying the formulas for pooling means and standard deviations of two groups, from the laboratory reference ranges for FGF23, and from unpublished data from another study (16 healthy men aged 22–54 years) for the RAAS components.*

*For Ang(1-7) 45 and 44 values were below the limit of quantification at baseline and end of study, respectively. None of the ACE2 were below detection limit.

*^†^Reported as mean and standard deviation.*

### Mediation of FGF23 via Renin-Angiotensin-Aldosterone System Parameters

We did not find an indication that the effect of FGF23 on LVH was mediated by the RAAS parameters. For all RAAS parameters, the estimated direct effects of FGF23 fold change were similar to the effect reported in Table 3 (differences only due to additional adjustment for a single RAAS parameter), while the indirect effects were always close to zero. Detailed numerical results are provided in Table 4. Additionally adjusting for the intake of RAAS medication did not affect these results (not shown).

**TABLE 3:** Association between FGF23 and RAAS parameters.

RAAS outcome (Log2 levels and 95%CI)	Log2 levels of FGF23		log2 FGF23 fold change per year	
		
	Association with baseline RAAS levels	Association with RAAS end of study levels	Association with RAAS end of study levels	Drop in R^2^ (relative to model R^2^)
ACE2	0.06 (−0.01, 0.12)	−0.01 (−0.08, 0.06)	0.01 (−0.08, 0.10)	0.00 (<1%)
Angiotensin II	−0.22 (−0.43, −0.02)	−0.04 (−0.22, 0.14)	−0.08 (−0.31, 0.14)	0.00 (2%)
Aldosterone	−0.05 (−0.30, 0.20)	−0.18 (−0.33, −0.04)	−0.13 (−0.31, 0.05)	0.02 (5%)

*No strong associations were detected. Coefficients reported are to be interpreted as follows: if the levels of FGF23 or FGF23 fold change double, then the outcome is multiplied by the factor 2^coefficient. Numbers report the regression coefficients (95% CI) for the association of FGF23 levels and FGF23 fold change per year obtained by simple linear regression models for the log2-transformed levels for RAAS parameters at baseline and end of study. Models for baseline (pre-treatment) included the pre-treatment levels of FGF23 only, models for end of study additionally adjusted for pre-treatment levels of the RAAS outcome. Models comprising the FGF23 fold change per year were also adjusted for pre-treatment levels of the RAAS outcome. Thus, if coefficients are larger than 0, then outcome levels increase, otherwise they decrease per doubling of the exposure variable. Ninety five percentage confidence intervals for the FGF23 fold change models were obtained from 1,000 bootstrap resamples.*

*Drop in R^2^ indicates the drop in the amount of explained variation of the outcome when FGF23 fold change is removed from the model. Results for aldosterone were strongly affected by removal of values below LLOQ, as reported in Supplementary Table 1.*

**TABLE 4:** Estimated direct and mediated effects of FGF23 on left ventricular hypertrophy (LVH) for each of the renin-angiotensin-aldosterone system (RAAS) parameters of interest.

RAAS parameter used as potential mediator	Direct effect of FGF23 on LVH (95% CI)	Indirect effect of FGF23 on LVH via RAAS parameter (95% CI)
ACE2	2.5 (1.1, 4.1)	0.0 (−0.2, 0.3)
Angiotensin II	2.8 (1.7, 4.2)	−0.2 (−1.0, 0.7)
Aldosterone	2.4 (1.3, 3.8)	−0.4 (−1.2, 0.2)

*The direct effect was estimated in a model for the change in LVMI including the change in a single RAAS parameter as mediator and FGF23 fold change as main exposure. The indirect effect was estimated using the product method for mediation and used a second model for the corresponding RAAS parameter including FGF23 fold change as exposure. The indirect effect was then given as the product of the effect estimates from both models. All models used the randomization factors (study center, residual kidney function), as well as corresponding baseline RAAS and FGF23 levels as covariates. Confidence intervals were computed using 1,000 bootstrap resamples.*

### Association of FGF23 With Renin-Angiotensin-Aldosterone System Parameters

**FIGURE 3:**
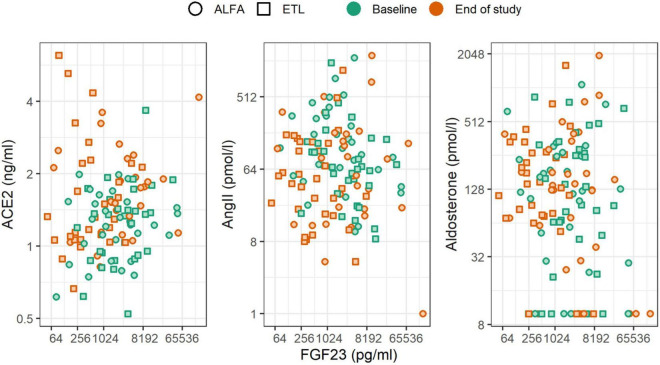
Scatterplot of FGF23 levels and RAAS parameters stratified by the time of measurement (color) and study drug (shape). Changes of FGF23 were not associated with changes of RAAS parameters.

In general, we found no associations of FGF23 levels and RAAS levels at baseline and end of study. Neither did we observe an association of FGF23 changes during the study and RAAS levels at the end of the study (Figure 3 and Table 3). The scatterplots show no distinct patterns for baseline and end of study levels, which was corroborated by the analyses using linear regression. All of the estimated effects have magnitudes close to zero and 95% CIs including zero. Similarly, no association between the change in FGF23 during the study, in terms of fold change per year, and the RAAS parameters was observed. This is also evident from the low drop-in-R^2^ values, which are all virtually zero. Inclusion of the study drug as adjustment variable did not influence the findings, and the same applies to the additional covariate representing treatment with RAAS inhibitors throughout the study (data not shown). For aldosterone the imputation of values below the LLOQ led to an increased effect magnitude, which diminished when these values were removed from the dataset (compare Table 3 with Supplementary Table 1). Furthermore, there were no associations of parathyroid hormone levels and RAAS levels at baseline and end of study.

### Association of Renin-Angiotensin-Aldosterone System Parameters and FGF23 With Left Ventricular Mass Index

**FIGURE 4:**
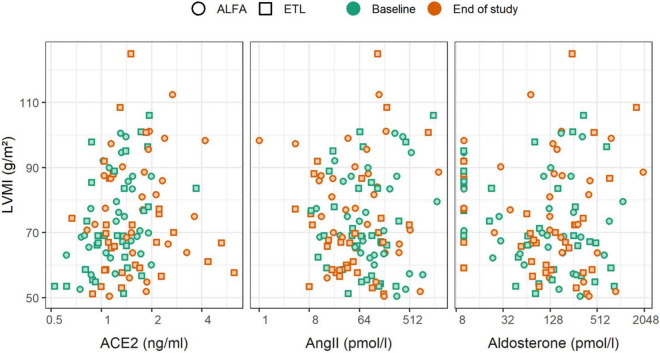
Scatterplot of renin-angiotensin-aldosterone system (RAAS) parameters and left ventricular mass index (LVMI), stratified by time of measurement (color) and study drug (shape). Changes of RAAS parameters were not associated with changes of LVMI.

In general the association between RAAS parameters and LVMI change was negligible, while FGF23 showed a statistical significant association with LVMI change. Figure 4 depicts the relationship graphically, while Table 5 reports the results from linear regression models. The strongest trend for LVMI change was observed for aldosterone, but the result was largely influenced by the presence of imputed values below the LLOQ. This is evident by the comparison with the results in Supplementary Table 2, for which such values were removed from the analysis. A clear association of FGF23 levels and LVMI was found at the end of the study, and in the analysis of FGF23 fold change and LVMI change. FGF23 fold change per year had a larger amount of explained variability and effect magnitude (with a 95% CI excluding zero) than any of the RAAS parameters. This association remained stable, even when including each of the RAAS parameters as covariate (data not shown). This finding further corroborated that the effect of FGF23 on the outcome LVMI is not mediated through the investigated RAAS parameters. Neither the adjustment for study-treatment allocation nor adjustment for intake of RAAS inhibiting medication markedly modified the effects reported here (data not shown).

**TABLE 5:** Association of renin-angiotensin-aldosterone system (RAAS) parameters and FGF23 with left ventricular mass index (LVMI).

RAAS parameters (95% CI)	Association of log2 levels with LVMI (95% CI) [g/m^2^]		Association of log2 fold change per year with LVMI change (95% CI)* [g/m^2^]	
		
	Association at baseline	Association at end of study	Association	Drop in R^2^ (relative to total R^2^)
ACE2	10.9 (3.8, 18.0)	0.2 (−4.5, 5.0)	−0.5 (−6.1, 5.1)	0.06 (38%)
Angiotensin II	0.51 (−1.7, 2.7)	−1.0 (−2.5, 0.5)	−1.1 (−2.9, 0.7)	0.04 (30%)
Aldosterone	−1.5 (−3.3, 0.4)	−0.3 (−1.9, 1.3)	−2.4 (−4.3, −0.42)	0.14 (56%)
FGF23	0.3 (−1.6, 2.1)	2.4 (1.3, 3.4)	2.6 (1.6, 3.9)	0.22 (80%)

*The reported coefficients are to be interpreted as follows: per doubling of the levels of the specific RAAS parameter, the outcome (LVMI levels or LVMI change) changes by the value of the coefficient. Numbers report the regression coefficients (95% CI) for the association of RAAS components or FGF23 levels, as well as corresponding fold change per year obtained by simple linear regression models for the log2-transformed levels for LVMI at baseline and end of study, or LVMI change (defined as levels at end of study minus levels at baseline). Results for aldosterone were strongly affected by removal of values below LLOQ, as reported in Supplementary Table 2. Due to our trial design for the comparison of the two study medications modifying FGF23, results for the crude, unadjusted baseline associations may not be necessarily indicative of an absence of association due to high variability of the exposures, and potential confounding for observed baseline outcome measurement. This potential confounding is controlled for by the design in the end of study and change analyses.*

*Adjusted for randomization factors (dialysis center, residual kidney function) and baseline levels of LVMI. For FGF23 fold change per year the 95% CI was obtained from 1000 bootstrap resamples.

## Discussion

In the present study we found that systemic RAAS activity was not increased in hemodialysis patients and remained largely unaffected by changes in FGF23 levels caused by ETL or alfacalcidol treatment, and is unrelated to LVMI.

Large trials have shown that RAAS inhibiting medication reduces LVH and improves patient survival in the general population independent of blood pressure reduction (4, 26). Cardioprotective effects are mainly attributed to a reduction of AngII-induced myocardial hypertrophy and of fibrosis (27, 28). However, despite the frequent use of RAAS-blocking medication also in end-stage kidney disease, their beneficial cardiovascular effect in this patient cohort remains unclear. Neither of the randomized placebo-controlled studies OCTOPUS or SAFIR showed a lower risk of cardiovascular events through the use of angiotensin receptor blockers among patients on hemodialysis (29, 30). The literature on the effect of RAAS inhibition on LVMI in hemodialysis is conflicting. In a trial by Hammer et al. (31) 97 hemodialysis patients were randomized to receive either spironolactone or placebo for a duration of 40 weeks. They were unable to show an effect of the treatment on LVH. Yu et al. (32) presented no significant regression of LVH in hemodialysis patients after 12 months of ramipril treatment. On the other hand, a meta-analysis by Tai et al. (33) showed a LV mass reduction through treatment with ACE inhibitors or angiotensin receptor blockers in the same patient collective. In these trials pre-existing LVH was not an inclusion criterion. In addition, with the exception of the MiREnDA trial, LV mass was quantified using echocardiography, which has a high inter- and intra-observer variability as compared to CMR (34). Furthermore, in all studies treatment was provided orally, risking possible patient non-adherence.

The activation of the RAAS in CKD patients not on dialysis results from both renal ischemia as well as volume retention, which both stimulate renin release from the juxtaglomerular apparatus (35). RAAS blocking medications are well-established treatment options preventing both CKD progression and premature mortality (36, 37). Interestingly, in our cohort of hemodialysis patients, plasma renin activity, AngII and aldosterone were lower compared to healthy individuals, potentially explaining the limited effect of RAAS blocking medication in this patient collective. This is in line with previous data in end-stage kidney disease patients (38). It is important to point out, that the healthy participants consisted of merely male controls, who were younger than our study cohort (between 22 and 54 years) and not taking antihypertensive drugs. Therefore, it would have been interesting to also be able to compare the levels of RAAS parameters to non-CKD individuals under RAAS inhibitors.

Interestingly, the PRA-S levels were reported to be even lower in anephric patients while AngII and aldosterone levels were within the normal range as reported by Liu et al. (39). A trial by Wilkes et al. (40) reported almost undetectable renin levels and suppressed AngII in anephric patients which were uninfluenced by volume reduction through hemodialysis treatment. Both Liu and Wilkes used radioimmunoassays for the analysis of RAAS parameters, while we made use of mass spectrometry-based quantification, making a direct comparison of results difficult.

In contrast to RAAS inhibition, pharmacological reduction of FGF23 levels significantly reduced LV mass in hemodialysis patients. A molecular interplay between FGF23 and RAAS was previously reported in CKD stage 1–5, but we did not find an association between serum levels of the two regulatory systems in our patients on maintenance dialysis (9). The mentioned study presenting an interplay between FGF23 and aldosterone across four species served as a scientific hypothesis that made us assume a similar finding in our patient collective (16). However, it is important to point out, that the humans and animals analyzed in this trial were CKD stage 1–5, but not end-stage kidney disease. Likewise, a previously performed cross-sectional study in 180 CKD patients found a positive association between FGF23 and aldosterone levels. Once again, the studied subjects were non-dialysis CKD patients (41). Our data suggest that LV remodeling in hemodialysis patients may be different from the general population and primarily dependent on FGF23 metabolism rather than systemic RAAS activation. Potential changes in local RAAS regulation contributing to myocardial hypertrophy and fibrosis were not analyzed in our study (42, 43). Measurements of tissue RAAS parameters would necessitate a more invasive collection of samples.

Limitations of our study include a limited power to detect and estimate effects of FGF23 on RAAS parameters, and their association with LVMI. However, our study data suggests that such effects, if present, are likely not very large. Subjects were not stratified for the intake of blood pressure medication influencing the RAAS. However, the proportion of patients under this medication was similar in both treatment groups and RAAS parameters were equally low in the 58% of patients without RAAS blockade (15). Furthermore, the samples were drawn at different times during the day depending on the starting time of hemodialysis (morning, noon or evening), which is important to point out because RAAS parameters are influenced by circadian rhythm (44). However, RAAS parameters did not differ between morning, noon and evening samples and each individual patient usually received treatment at the same time of the day.

The main strengths of our study include the prospective design as well as the completed follow-up. The use of intravenous treatment enabled a reliable and constant delivery of the study drug. Furthermore, LVMI was analyzed using CMR, which is the gold standard imaging technique for the quantification of LVH and observer independent. In order to minimize confounding of LVMI changes by pressure and volume overload, which are the main drivers of LVH in dialysis patients, the subjects were carefully evaluated for blood pressure and body fluid composition, as well as stratified for residual urine output. Blood pressure was measured before, during and after each dialysis session and body fluid composition was evaluated using body composition monitoring based on bioimpedance spectroscopy during screening and throughout the study every 2 months (45). As reported previously, both the blood pressure and use of antihypertensive medication were comparable between treatment groups (15). The measurements of RAAS parameters were performed using state of the art mass spectrometry. All samples were collected at the beginning of hemodialysis treatment after patients lay in supine position for several minutes, which is important considering that angiotensin metabolites are hormones which are dependent of body position and volume status. The latter was repeatedly controlled during the trial using body composition monitoring.

In summary, we found that systemic RAAS parameters were not elevated in hemodialysis patients possibly providing a potential mechanistic explanation for the reduced efficacy of RAAS blocking medication to prevent LV hypertrophy in hemodialysis patients. A clearer statement about the effectiveness of this medication in this specific patient cohort would require a much larger sample size. However, FGF23 serum level trajectories modifiable by treatment with ETL were associated with the progression of LVMI, and we found no indication of mediation via systemic RAAS. This suggests a different mechanism of LVH in hemodialysis patients compared to the general population.

## Data Availability Statement

The original contributions presented in the study are included in the article/Supplementary Material, further inquiries can be directed to the corresponding author.

## Ethics Statement

The studies involving human participants were reviewed and approved by Ethics committee of the Medical University of Vienna (MUV; [EK # 1127/2017]). The patients/participants provided their written informed consent to participate in this study.

## Author Contributions

KD, RO, RR-S, and MK were responsible for the conceptualization and methodology of the trial. KD was tasked with the investigation, including data collection, with support by ML. MK was assigned with the formal analysis and software. DB performed the analysis of MRI data. RM and MP were responsible for laboratory analysis. Writing of the original draft was performed by KD and reviewed and edited by MK, RR-S, ML, RM, MP, DB, and RO. All authors contributed to the article and approved the submitted version.

## Conflict of Interest

RO reported grants from Amgen during the conduct of the study. In addition, RO, KD, and RR-S had a patent “Methods of treating left ventricle hypertrophy” pending. MP was employed by Attoquant Diagnostics. The remaining authors declare that the research was conducted in the absence of any commercial or financial relationships that could be construed as a potential conflict of interest.

## Publisher’s Note

All claims expressed in this article are solely those of the authors and do not necessarily represent those of their affiliated organizations, or those of the publisher, the editors and the reviewers. Any product that may be evaluated in this article, or claim that may be made by its manufacturer, is not guaranteed or endorsed by the publisher.

## Abbreviations

AngII: angiotensin II
ACE2: angiotensin-converting enzyme-2
CKD: chronic kidney disease
CMR: cardiac magnetic resonance imaging
EtECAR-HD: effect of etelcalcetide on cardiac hypertrophy in hemodialysis patients–a randomized controlled trial
ETL: etelcalcetide
FGF23: fibroblast growth factor 23
LVH: left ventricular hypertrophy
LVMI: left ventricular mass index
MUV: Medical University of Vienna
PRA-S: plasma renin activity-surrogate
RAAS: renin-angiotensin-aldosterone system.
