# Supplementary material for: The Effect of FGF23 on Cardiac Hypertrophy Is Not Mediated by Systemic Renin-Angiotensin- Aldosterone System in Hemodialysis
Source: Front Med (Lausanne). 2022 Apr 26;9:878730. doi: 10.3389/fmed.2022.878730 (PMC9086596; doi:10.3389/fmed.2022.878730)
Supplement: Supplementary file 1 [file Data_Sheet_1.docx]

Supplementary Material

# Supplementary Figures and Tables

# 1. Supplementary Figures


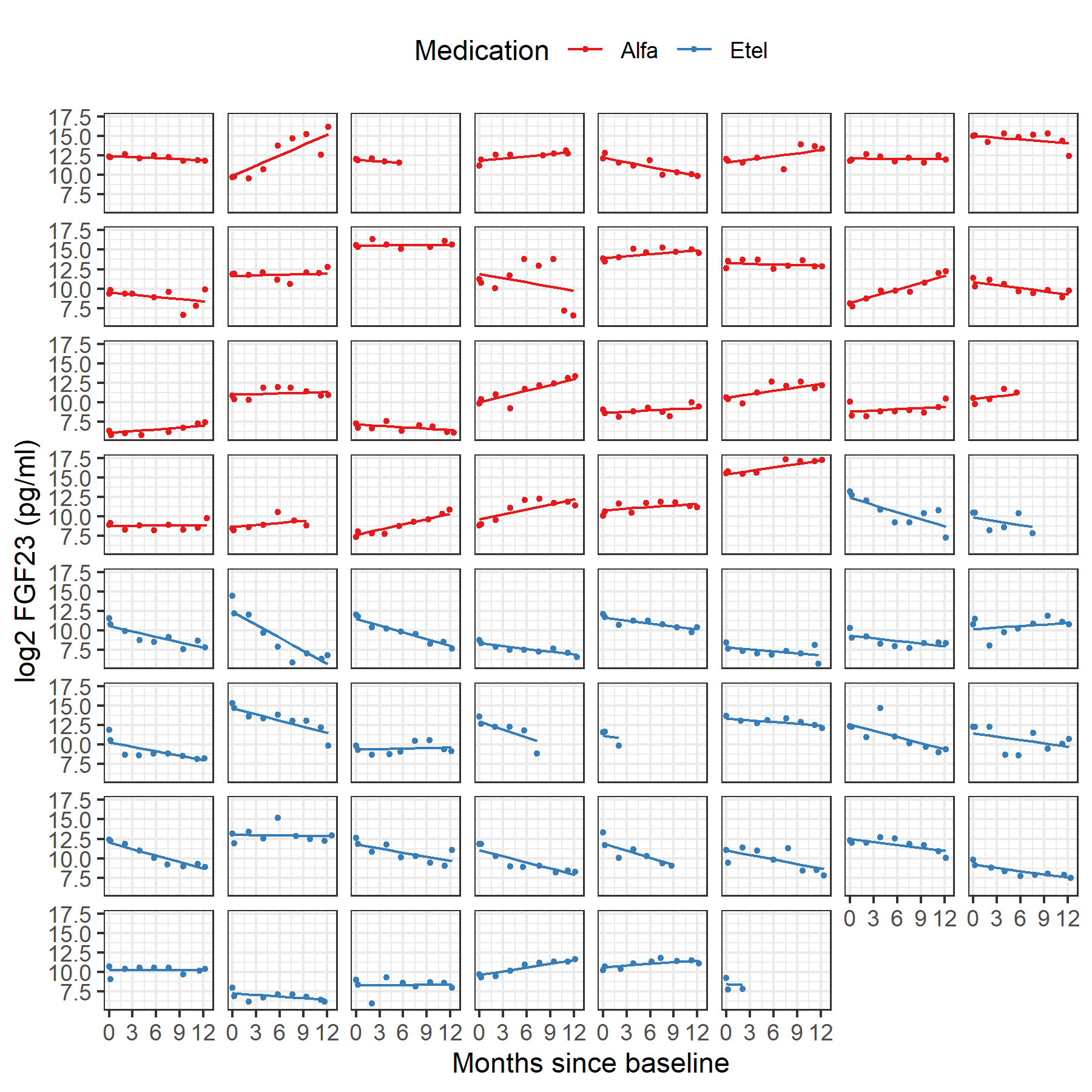


Supplementary Figure 1**:**

Mixed model estimates of trajectories of FGF23 levels throughout the

study. The graphic depicts individual measurements of FGF23 levels throughout the study as

points. Each panel depicts one individual of the study (62 in total). The colors indicate the

study drug an individual was randomized to. The solid line was derived from a linear mixed

model by the summation of the fixed intercept and effect of time with the random intercepts

and random effects of time per individual, respectively.

Generally, the measurements are in good agreement with the predicted values, with the model

(including random effects) explaining 87% of the variation in the observed FGF23 levels.

# Supplementary Tables

Supplementary Table 1: Association of FGF23 and aldosterone with values below the LLOQ excluded (ACE2 and AngII results were identical to the main results).

| **RAAS outcome**  **(Log2 levels)** | **Log2 levels of FGF23** | | **log2 FGF23 fold change per year** | |
| --- | --- | --- | --- | --- |
|  | **Association with baseline**  **RAAS levels** | **Association with RAAS end of study levels** | **Association with RAAS end of study levels** | **Drop in R² (relative to model R²)** |
| Aldosterone | -0.06  (-0.26, 0.14) | 0.05  (-0.12, 0.22) | 0.00  (-0.12, 0.23) | 0.01 (6%) |

Results shown are the regression coefficients (95% CI) for the association of FGF23 levels and FGF23 fold change per year obtained by simple linear regression models for the log2-transformed levels for RAAS parameters at baseline and end of study. Models for baseline (pre-treatment) included the pre-treatment levels of FGF23 only, models for end of study additionally adjusted for pre-treatment levels of the RAAS outcome. Models comprising the FGF23 fold change per year were also adjusted for pre-treatment levels of the RAAS outcome. Coefficients reported are to be interpreted as follows: if the levels of FGF23 or FGF23 fold change double, then the outcome is multiplied by the factor 2^coefficient. Thus, if coefficients are large than 0, then outcome levels increase, otherwise they decrease per doubling of the exposure variable. 95% confidence intervals for the FGF23 fold change models were obtained from 1000 bootstrap resamples. Drop in R² indicates the drop in the amount of explained variation of the outcome when FGF23 fold change is removed from the model.

Supplementary Table 2**:** Association of aldosterone excluding values below the LLOQ and LVMI (ACE2 and Angiotensin II results were identical to the main results).

| **RAAS parameter** | **Association of log2 levels with LVMI levels (95% CI)** | | **Association of log2 fold change per year with LVMI change (95% CI)*** | |
| --- | --- | --- | --- | --- |
|  | **Association at baseline** | **Association at end of study** | **Association** | **Drop in R² (relative to total R²)** |
| Aldosterone | 0.6  (-2.3, 3.6) | 0.7  (-1.7, 3.1) | -1.0  (-3.5, 1.5) | 0.1 (49%) |

The reported coefficients are to be interpreted as follows: per doubling of the levels of the specific RAAS parameter, the outcome (LVMI levels or LVMI change) changes by the value of the coefficient.
* adjusted for randomization factors (dialysis center, residual kidney function) and baseline levels of LVMI.

Supplementary Table 3: Values below lower limit of quantification for RAAS parameter.

| **RAAS parameter** | **LLOQ*** | **Baseline** | **End of study** |
| --- | --- | --- | --- |
| PRA-S | 5 | 0 (0%) | 0 (0%) |
| ACE2 | 0.5 | 0 (0%) | 0 (0%) |
| Ang(1-7) | 3 | 45 (73%) | 44 (71%) |
| Angiotensin II | 2 | 0 (0%) | 1 (2%) |
| Aldosterone | 20 | 11 (18%) | 9 (15%) |

* Lower limit of quantification
